# Attention mechanisms and the mosaic evolution of speech

**DOI:** 10.3389/fpsyg.2014.01463

**Published:** 2014-12-16

**Authors:** Pedro T. Martins, Cedric Boeckx

**Affiliations:** ^1^Department of Information and Communication Technologies, Pompeu Fabra University, Barcelona, Spain; ^2^Center of Linguistics of the University of Porto, Porto, Portugal; ^3^Biolinguistics Initiative Barcelona, Barcelona, Spain; ^4^Department of General Linguistics, University of Barcelona, Barcelona, Spain; ^5^Catalan Institute of Research and Advanced Studies (ICREA), Barcelona, Spain

**Keywords:** evolution of speech, attention mechanisms, consonants and vowels, evolution, oscillatory cycles

## Abstract

There is still no categorical answer as to why humans, and no other species, have speech, or why speech is the way it is. Several purely anatomical arguments have been put forward, but they have been shown to be false, biologically implausible, or of limited scope. This perspective paper supports the idea that evolutionary theories of speech could benefit from a focus on the cognitive mechanisms that make speech possible, for which antecedents in evolutionary history and brain correlates can be found. This type of approach is part of a very recent but rapidly growing trend that has already provided crucial insights on the nature of human speech by focusing on the biological bases of vocal learning. Here we contend that a general mechanism of attention, which manifests itself not only in the visual but also in the auditory modality, might be one of the key ingredients of human speech, in addition to the mechanisms underlying vocal learning, and the pairing of facial gestures with vocalic units.

The mechanics of speech have been thoroughly studied. Various techniques and methodologies have been developed that allow us to know with great precision what goes on anatomically when human and non-human primates vocalize, from the lungs to the lips (Hardcastle et al., 1989; Fitch and Hauser, 1995; Fishman, 2003; Ghazanfar and Rendall, 2008). However, the question of *why* (only) humans have speech in the first place remains to be categorically answered. Different purely anatomical arguments have been put forward, such as the uniqueness of the descended human larynx (Fant, 1960; Lieberman and Crelin, 1971) or the loss of air sacs in humans (de Boer, 2012), but both arguments have been seriously questioned (Fitch and Reby, 2001; Nishimura et al., 2006; Littauer, 2012). In fact arguments of this type all share a general problem: they fail to grasp the mosaic nature of cognitive faculties that evolution has tinkered with. Modern evolutionary biology shows that complex traits—and surely speech or indeed language as a whole falls within that category—require complex and multi-dimensional explanations (West-Eberhard, 2003; Pigliucci and Müller, 2010).

In addition, there are cases of other, non-human, even non-vocal-learning species that are capable of producing human-like vowels (Vs) and consonants (Cs), such as the Gelada Baboons (*Theropithecus gelada*), which seem to possess an extremely rich sound repertoire, comparable to that of humans. More specifically, it has been shown that this species is able to produce vocalizations that not only employ what we would perceive as Cs and Vs, but also are structured in a way that resembles human sound systems, with different vowel qualities and Cs distinguished by manner and place of articulation, as well as duration similar to that of human speech (Richman, 1976, et seq; Bergman, 2013). There are, of course, different ways of articulating sounds with the same acoustic effect, even among humans, but the very fact that there are indeed other species that are able to produce Cs and Vs in a dynamic manner and yet lack human-like speech, shows that merely having that inventory is not a diagnosis for neither speech nor language. So why is it, then, that we humans have it and species like Gelada Baboons don’t? We agree with a growing trend in the study of human speech (Deacon, 1997; MacNeilage, 2008; Fitch, 2010) that the answer surely has to do with the presence of vocal learning mechanisms in humans, for which a biological basis is emerging (a robust direct laryngeal connection from the motor cortex seems to be key; Fitch, 2010). But we contend that answers currently entertained in the literature are insufficient to account for a foundational property of speech: its Consonant–Vowel-based *organization*. Other species, including vocal learning ones, do not organize their vocal behavior the way we do.

It is generally agreed upon that vowel and consonant sounds definitely exist. There are mechanical reasons for this (see, e.g., Fant, 1960; MacNeilage, 1998, among others). In linguistics, especially since the work of Chomsky and Halle (1968), it has been generally assumed that speech sounds are abstractly represented as bundles of features, which must be somehow encoded in the brain [see Bouchard et al. (2013) and Mesgarani et al. (2014) for recent brain work regarding the latter point. Whether features are innately specified or not is an issue that does not bear on what follows, and on which we take no stand; see Clements and Ridouane (2011) for discussion of different perspectives]. Whatever basic units of phonological analysis one chooses, they generally boil down to articulatory characteristics (e.g., “bilabial” or “voiced”). However, since just being able to perceive or produce Vs and Cs is not a diagnostic for speech, as the vocal behavior of the Geladas illustrates, the productive use of Cs and Vs in something like speech (and language) might be explained not only by their encoding in the brain, but also, and crucially, by their distinct *functional/cognitive values*.

It has been shown that Vs and Cs are not treated on a par by human brains. When presented with speech, humans not only pick out the segments that comprise the sound continuum, but also ascribe different functional/cognitive weight to different kinds of segments. Vs and Cs indeed have different roles, with Cs providing lexical cues and Vs providing cues about syntactic structures (Nespor et al., 2003). Following Toro et al. (2008), we will refer to this as the CV hypothesis. A common initial objection to the CV hypothesis as stated is that the reason why humans process Cs and Vs in this differentiated manner is their asymmetric statistical distribution. Languages usually have more Cs than Vs, and they are distributed in such a way as to facilitate the extraction of transitional probabilities of Cs and their lexical information, with the subtler alternations of Vs providing the cues for structural information. If this were true, humans would be able to extract lexical and structural information based on the statistical distribution of segments alone, regardless of their being Vs or Cs. Building on previous work (e.g., Bonatti et al., 2005), Toro et al. (2008) tested just that, and found that the bias is deeper than “mere” statistics: they inverted the roles of Cs and Vs in the data and presented it to several subjects, who were simply unable to extract the same rules from the signal. Another objection to the CV hypotheses would be that the acoustic differences between Cs and Vs are responsible for their differentiated processing. But if physical aspects of speech sounds were the sole responsible for rule extraction one should not expect variation in their functional roles based on whether the same sounds are interpreted as speech sounds or as noise. However, research points the other way. For example, language-related areas are modulated differently by identical sounds depending on whether they are perceived as speech or non-speech (Möttönen et al., 2006), and audio-visual speech perception is triggered by acoustic stimuli perceived as speech, and not triggered when the exact same stimuli are perceived as something else (Toumainen et al., 2005).

These results show that the claim that language acquisition is made possible by general-purpose learning mechanisms (e.g., Elman et al., 1996) must be qualified: surely, if this were the case, humans would have no problem extracting different kinds of information from any sound system with asymmetrically distributed segments. It seems instead that there is a more basic biological bias for extracting lexical information for Cs and structural information from Vs, by virtue of their functional—and not statistical—differences.

More generally, it is not the case that the human brain processes different information equally. Not unlike speech, vision is a good example of selective processing of noisy input. In a recent study (Fiebelkorn et al., 2013), researchers draw a very important connection between endogenous oscillatory rhythms and space-based and object-based selection mechanisms. They suggest that the problem of retrieving the right information despite the abundance of signal is achieved through “rhythmic patterns of visual-target detection both within (8 Hz) and between (4 Hz) objects” (p. 2553). Compatible results are reported by Landau and Fries (2012).

These frequencies fall right within the range reported in Giraud and Poeppel (2012): the articulatory and the auditory systems structure their outputs in agreement with one another, that is, they are mediated by something which allows them to be in sync. This infrastructure provided by neuronal oscillations might be the key in explaining how the brain decodes continuous speech. Crucially, there is a robust relation between the time scales associated with speech cues (phonemes, syllables, and intonational phrases) and the time constants underlying neuronal oscillations (low-gamma, theta, and delta oscillations). These same oscillatory cycles have been linked to various “putative precursors” of speech, such as monkey lip-smacking (Ghazanfar et al., 2012; Fitch, 2013). We contend that in fact these entrainment patterns, despite being manifested upon contact with different kind of stimuli, point to one very general attention mechanism, which has been put to new use in humans, and which has given speech one of its most distinctive signatures. Specifically, the fact that two different patterns (∼4 Hz and ∼8 Hz) are associated, respectively, with within- and between-object attention (Fiebelkorn et al., 2013), plausibly reveals that Cs and Vs are specifically targeted by these different frequencies, or at least by a low-frequency/high-frequency dichotomy within the ranges reported and reviewed by the studies cited above. This would help explain why Cs are associated with lexical properties (*between-*word) and Vs with syntactic/structural properties (*within-*word) in the sound continuum. This would represent a very important ingredient of human speech, absent in other species, including other vocal-learners. A central question for our proposal is whether this attention mechanism is confined to a single domain or, instead, much more general.

Close relationships have been drawn between the underlying mechanisms behind visual and auditory attention, which all revolve around the recognition, selection and processing of information in space and/or time. de Freitas et al. (2013) ran attention experiments on which they tested the so-called *same-object advantage* (when the same physical distance is considered, responses are faster when probes occur within the same object than when in other objects) in the sound domain. Indeed, they show that responses are also faster within the same rhythmic phrase (a tone of a single frequency) than across different rhythmic phrases, with duration being the analog of distance. These results strongly suggest that human object-based attention is not exclusive to vision, and most likely not fundamentally spatial, but rather shared across domains.

There is remarkable coherence between the acoustic and visual cues of speech, such as temporal correspondence between mouth opening and acoustic envelope, area of mouth opening and formants, and temporal modulation of mouth movements and voice envelope (2–7 Hz; Chandrasekaran et al., 2009). A popular topic that comes up when discussing the relation between visual and auditory speech cues is the McGurk effect (McGurk and MacDonald, 1976), but here we are referring to something different: while the McGurk effect refers to the interference of (discrepant) visual cues in acoustic perception (see Tiippana, 2014 for a clarification of some misconceptions in this regard), we instead refer to the shared history and interdependence of auditory and speech cues at the neural level. Indeed, speech rhythm and facial expressions in humans are both rhythmic (3–8 Hz) and very much correlated (Golumbic et al., 2013; Ghazanfar and Takahashi, 2014a,b). Such a correlation has moreover been deemed crucial for the social interaction required for speech to prosper, that is, the coordination of individuals of a group through the syncing of neural processes across brains, in what has been called “brain-to-brain coupling” (Hasson et al., 2012). As these authors argue, if cognitive processes underlying complex behavior depended solely on the processing within the individual’s brain, it would hard—if not impossible—to reach a set of rules for interactive behavior to follow and sync. By sending cyclic, brain-generated signals through the physical environment to another brain, which decodes and accommodates them, brains really do sync through oscillatory activity.

On the basis of the findings we have pointed out so far, it is plausible that this mechanism is indeed general, thus representing a good example of an already existing capacity put to new use. Presumably, the recruitment of this domain-general attention mechanism in the domain of speech was the solution to the externalization of the complex syntactic/semantic component that other species lack (Berwick et al., 2011).

A prediction of our hypothesis is that non-human animals—crucially those which have been shown to distinguish between Vs and Cs, which might or might not be able to produce them—will display no functional difference between these two kinds of segments, and whatever rules they extract from auditory input will therefore not depend on the cues being Vs or Cs. This is also a prediction of de la Mora and Toro (2013), who performed experiments on Long–Evans rats (*Rattus norvegicus*) and concluded that they actually surpass humans in rule extraction from auditory input tasks: whereas rats had no problem generalizing rules in CVCVCV words both over Vs and Cs, humans could only do it for the Vs, with the same stimuli. Further rule-extracting experiments with non-human animals will surely strengthen the import of these results.

Though related, de la Mora and Toro’s (2013) prediction and ours, however, are not equivalent. For them, whatever makes us worse than mice at rule extraction from auditory data and thus better than them at inferring lexical and structural information must be unique to language, and to humans [by definition, this would fall under “Faculty of Language in the Narrow Sense” (Hauser et al., 2002)]. They leave the exact nature of this constraint up for grabs, but they assume that only “if the observed differences in how humans process speech are a result of language-specific constraints, we should not observe functional differences in other species.” (p. 308) Our prediction makes no claim of uniqueness to language; we contend that the functional difference humans attribute to Cs and Vs is due to non-linguistic aspects of our neurology, namely a general mechanism of attention, and which along with vocal learning and the ability to produce a large enough sound inventory formed the basis of human speech. We agree with Gervain and Mehler (2010, p. 196), that “[i]f humans and non-human animals share cognitive and/or learning abilities, these cannot be language specific since only our species have language. However, they may have been precursors bringing humans closer to language.”

Our perspective may also benefit from a closer examination of the relation between the attention mechanism appealed to here and the general issue of working memory. The literature on working memory often relates to the one on attention. Furthermore, it is known that the storage of Vs and Cs differ, both in terms of stability (storing of Vs being more stable than that of Cs), and in terms of sensitivity to order information (Vs being more related to order information in the phonological sequence than Cs; Drewnowski, 1980; Baddeley, 2007). This seems to converge with the proposal of Nespor et al. (2003), and with our emphasis on Cs and Vs having distinct cognitive imports.

A rapprochement between our proposal and working memory could shed light on the neuroanatomical basis of the C/V distinction, given recent progress in the characterization of human-specific connectivity patterns (see Aboitiz, 2012; Scott et al., 2012; Neubert et al., 2014, among others). We leave a detailed exploration of this issue for future research.

What is clear to us already is that mechanisms of the sort we have appealed to here are very much line with what de Waal and Ferrari (2010) call the bottom-up perspective on human and animal cognition: looking for wide-ranging, basic mechanisms across species and domains, instead of asking what is special and unique about any one trait and species.

As put by Fitch (2013, p. 27), “Although language, as a composite system, is clearly unique to our species, substantial empirical work is still required before any of the mechanisms involved in language can be conclusively labeled unique.” We think this true also of speech, and believe that the most fruitful way of unveiling its nature is to study the structure and evolution of each of the mechanisms involved, such as the one we put forth.

## AUTHOR CONTRIBUTION

Both authors contributed to the writing of this paper.

### Conflict of Interest Statement

The authors declare that the research was conducted in the absence of any commercial or financial relationships that could be construed as a potential conflict of interest.
